# Single-character insertion–deletion model preserves long indels in ancestral sequence reconstruction

**DOI:** 10.1186/s12859-024-05986-1

**Published:** 2024-12-02

**Authors:** Gholamhossein Jowkar, Jūlija Pečerska, Manuel Gil, Maria Anisimova

**Affiliations:** 1 Institute of Biology, University of Neuchâtel, Rue Emile-Argand 11, 2000 Neuchâtel, Neuchâtel Switzerland; 2 Swiss Institute of Bioinformatics, Quartier Sorge - Batiment Amphipôle, 1015 Lausanne, Vaud Switzerland; 3 Institute of Computational Life Sciences, School of Life Sciences and Facility Management, Zurich University of Applied Sciences (ZHAW), Schloss, 8820 Wädenswil, Zürich Switzerland

**Keywords:** Ancestral sequence reconstruction, Insertion, Deletion, Indel pattern, Long indel, Gap length distribution, Mammalian genomics, Poisson indel process

## Abstract

Insertions and deletions (indels) play a significant role in genome evolution across species. Realistic modelling of indel evolution is challenging and is still an open research question. Several attempts have been made to explicitly model multi-character (long) indels, such as TKF92, by relaxing the site independence assumption and introducing fragments. However, these methods are computationally expensive. On the other hand, the Poisson Indel Process (PIP) assumes site independence but allows one to infer single-character indels on the phylogenetic tree, distinguishing insertions from deletions. PIP’s marginal likelihood computation has linear time complexity, enabling ancestral sequence reconstruction (ASR) with indels in linear time. Recently, we developed ARPIP, an ASR method using PIP, capable of inferring indel events with explicit evolutionary interpretations. Here, we investigate the effect of the single-character indel assumption on reconstructed ancestral sequences on mammalian protein orthologs and on simulated data. We show that ARPIP’s ancestral estimates preserve the gap length distribution observed in the input alignment. In mammalian proteins the lengths of inserted segments appear to be substantially longer compared to deleted segments. Further, we confirm the well-established deletion bias observed in real data. To date, ARPIP is the only ancestral reconstruction method that explicitly models insertion and deletion events over time. Given a good quality input alignment, it can capture ancestral long indel events on the phylogeny.

## Introduction

Insertion and deletion (indel) events produce significant amounts of natural variation in species genomes. Consequently, indels make a major contribution to complex evolutionary processes. Today indel variants in genomic sequences can be reliably documented and studied due to improvements in sequencing methods. In closely related species, differences attributed to indels (per base pair) are several-fold more frequent than substitution events [1, 2]. In the human genome, up to a quarter of all genomic variants are due to indels, most of which are very short [3]. While indels are distributed across both coding and non-coding parts of genomes, they are far more frequent in non-coding sequences. Compared to substitutions, indel changes are expected to have a stronger deleterious effect on functional proteins [4], also explaining their lower prevalence in coding sequences. Despite this, many deleterious coding indel variants persist in the human population and can cause disease-related gene defects (e.g., [5]).

In comparative studies of sequence evolution, indels are represented as gaps in alignments of homologous sequences. With growing evolutionary distance, different indel events can merge and overlap, masking the mutational history. Nevertheless, alignment gaps carry much phylogenetic information [6], which can provide valuable insights for evolutionary studies when analyzed correctly. However, properly modelling the evolutionary process of insertions and deletions is challenging from the computational and modelling perspective, and there is no gold standard in the field. In fact, many evolutionary studies either completely ignore indels or heavily trim indel-rich sequence regions due to the lack of software tools implementing appropriate models. Disentangling individual insertion and deletion events based on the observed gap distributions in a multiple sequence alignment (MSA) requires modelling sequence evolution in a way that includes the insertion and deletion processes. One way to handle this is to employ fast parsimony-based approaches (e.g. Chindelevitch et al. [7], Iglhaut et al. [8]) to reconstruct indel histories. While powerful, these methods lack an explicit evolutionary model and, therefore, cannot infer event rates, meaning that the conclusions that can be made from these methods are limited. In this paper, we focus on investigating the reconstructing power of a probabilistic model of sequence evolution that includes the insertion and deletion processes over time, which can allow us to compare insertion, deletion and substitution rates in more general evolutionary contexts. Substitutions are traditionally described via Markov models assuming site independence, while indels violate this assumption since each indel event can involve multiple residues. Therefore, models that properly include these events tend to be computationally expensive.

The first evolutionary model with indels, TKF91, lifted the assumption of site independence and described single-character indels via a birth-death process [9]. As TKF91 models single-character events, it implies a linear gap cost in the MSA inference, but due to the non-independence of sites, the complexity of computing the marginal likelihood under this model is exponential in the number of taxa, making the basic tasks of phylogenetic inference (MSA and tree estimation) intractable. ASR under this model is also non-trivial, and while attempts have been made to develop a computationally tractable ancestral state estimator (e.g. Fan and Roch [10]) under this model, no methods implementing it exist at this point. Bouchard-Côté and Jordan [11] proposed the PIP model, a close relative of TKF91, where insertions follow the Poisson process while deletions are added to the Markov substitution model as an absorbing state. The complexity of marginal likelihood computation under the PIP model is reduced to linear, which allows for this model to be adopted for phylogenetic inferences [12–14]. Moreover, the formulation of the PIP likelihood makes reconstructing most likely indel histories possible in linear time as well [15]. However, like TKF91, PIP explicitly models only single-character indels.

Modelling longer indels as several independent single-character events lacks biological realism and could lead to biases such as homology histories with too many events, alignments with scattered gaps, and high indel rates. Some evolutionary indel models allow long indels [16–18]. For example, the TKF92 model, an extension of TKF91, is also a birth-death process but with indels happening as unbreakable multiple-site fragments with a geometric length distribution [16]. This modelling assumption, however, means that TKF92 cannot explain overlapping indels. The “long indel” model [17] relaxed the unbreakable fragment assumption but assumed infinite sequences. Both these models can be considered an approximation of the Generalised Geometric Indel (GGI) model [19]. However, while the lengths of individual indels have a geometrical distribution, the length distribution of observed gaps in the alignment is not geometric in general. Considering that models with long indels also tend to be computationally slow, these are currently of little practical value for large datasets.

Computationally, PIP holds promise for practical phylogenetic analyses despite the single-character indel assumption. For example, we showed that PIP-based alignment inference can pick up multiple-character indels (long indels) when the data strongly suggests this [13, 14]. Zhai and Bouchard-Côté [12] demonstrated that modelling indel evolution and indel rate variation improves the accuracy of phylogeny reconstruction when using the PIP model and its generalizations.

Recently, we proposed a PIP-based ancestral sequence reconstruction (ASR) approach implemented in ARPIP [15]. Apart from Bayesian MCMC implementations (e.g., Historian [20]), ARPIP is the only ASR method that uses an explicit model of indel evolution and can infer the specific locations of insertions and deletions on the tree. Another popular ASR method is FastML-webserver [21], which uses the so-called “indel-coding” method to include indels. This approach does not include a proper statistical model of insertion and deletion and implies that a deleted character can be reinserted. GRASP [22], another recent method, accommodates indels in the ASR inference by representing sequences as partial order graphs. However, as with indel-coding, deleted characters can be reinserted, and there is no explicit model governing the indel process.

## The goals of this study

Having an explicit model of indel evolution is desirable; however, an over-simplistic model could also have a detrimental effect on the resulting inferences, including overestimation of indel rates and scattered ancestral sequence alignments by including too many single-character gaps. Therefore, we aim to investigate whether using the single-character indel assumption negatively impacts ASR. Since ASR methods typically take a fixed MSA and phylogeny as input, using good-quality input MSAs and phylogenetic trees is imperative for accurate ASR, irrespective of the method used. While MSA quality is still quite an elusive concept in general, here we assume that a good-quality MSA captures multiple-character (long) indels in a phylogenetically consistent way. Therefore, in our study, we use PRANK [23], the phylogeny-aware tool which infers phylogenetically meaningful gaps by distinguishing insertions from deletions in a progressive manner on the tree.

Here, given accurate input data, we assess the systematic bias in PIP-based ASR by investigating the fragmenting of gaps in the inferred sequences at the ancestral nodes of the phylogeny. To test this, we present a large-scale analysis of protein orthologs from six mammalian species (human, three primates, and two rodents), taken from the popular orthologous protein database OMA [24], as well as analysis of simulated data. We chose this specific phylogenetic dataset for two reasons. First, the mammalian species tree for these specific taxa is unambiguous and can be accepted as “true” (although the indel history is unknown, see [25]). Second, insertion and deletion biases in these species have long been a subject of interest, meaning that our findings can be interpreted in the context of current literature. For these data, we evaluated per-site insertion and deletion frequencies in different lineages and compared the gap distributions in the observed and inferred sequences.

To get a better understanding of ASR properties and potential biases under PIP, we proceed by analyzing simulated data. In our simulations, we mimic the OMA-based protein orthologous groups so that the results on real data can be compared to expected performance on very similar data where the truth is known. Our results suggest no significant difference in observed and inferred ancestral gap length distributions. This means that ARPIP tends to preserve the long indels from the input alignment in the inferred ancestral sequences. We also could confirm the well-documented deletion bias [26–31].

## Results

### Results on mammalian data

We extracted and analyzed $12'022$ orthologous protein groups, each containing one sequence from six *eutherian* mammals. We filtered out the datasets for which the gene and species tree topologies agree to ensure that indel events can be meaningfully mapped to a common topology, which left us with $3'906$ datasets. Sequences in each orthologous group were aligned, and ancestral sequences were reconstructed given the inferred multiple sequence alignment (MSA) and the species tree (see data and methods). For each site in an MSA, our ASR method ARPIP infers the most likely insertion and deletion history, allowing us to distinguish insertion and deletion events. Note that the reconstruction is done independently for each site, as in all other ASR methods. Therefore, we evaluated the number of inserted and deleted residues per site and per time interval rather than counting multiple residue events. This way of measuring indel rates is intuitively similar to substitution rates; therefore, it has a simple interpretation without having to account for the length of the full indel. Another advantage of this approach is that it makes it easy to evaluate the impact of indel events on sequence length over time.

Note that we clearly distinguish between gaps and indels. Gaps are stretches of missing characters (gap characters “-”) that can either represent characters that existed in a lineage ancestral to the one in question and got deleted or characters inserted in a sister lineage, i.e. characters that never existed in the lineage in question. An insertion appears as a gap stretch in all lineages that do not belong to the clade where the insertion happened, meaning that the length of said stretch approximates the length of the inserted fragment. A deletion appears as a gap stretch in all lineages descendant from the one in which the deletion happened, meaning that the length of that stretch approximates the length of the deleted fragment. Both leaf and internal node sequences can contain both types of gap stretches; thus, all nodes in the tree can have indel events. However, the MSA defines the gap characters in the leaf nodes, while the ancestral gap characters are inferred with ASR. On the other hand, ASR attributes the event type (insertion or deletion) to gaps in both leaves and internal nodes.

Moreover, gaps in MSAs can appear due to several multiple-character insertions and deletions. Since ASR is performed independently at each site and PIP only accounts for single-character events, gaps spanning multiple sites are described as a series of single-character indel events at several affected individual sites. To evaluate whether this assumption is reasonable during ASR, we study whether the ARPIP method preserves the distribution of gap lengths of the input MSA in the sequences reconstructed at ancestral nodes.

#### Comparing the number of inserted and deleted characters

**Fig. 1:**
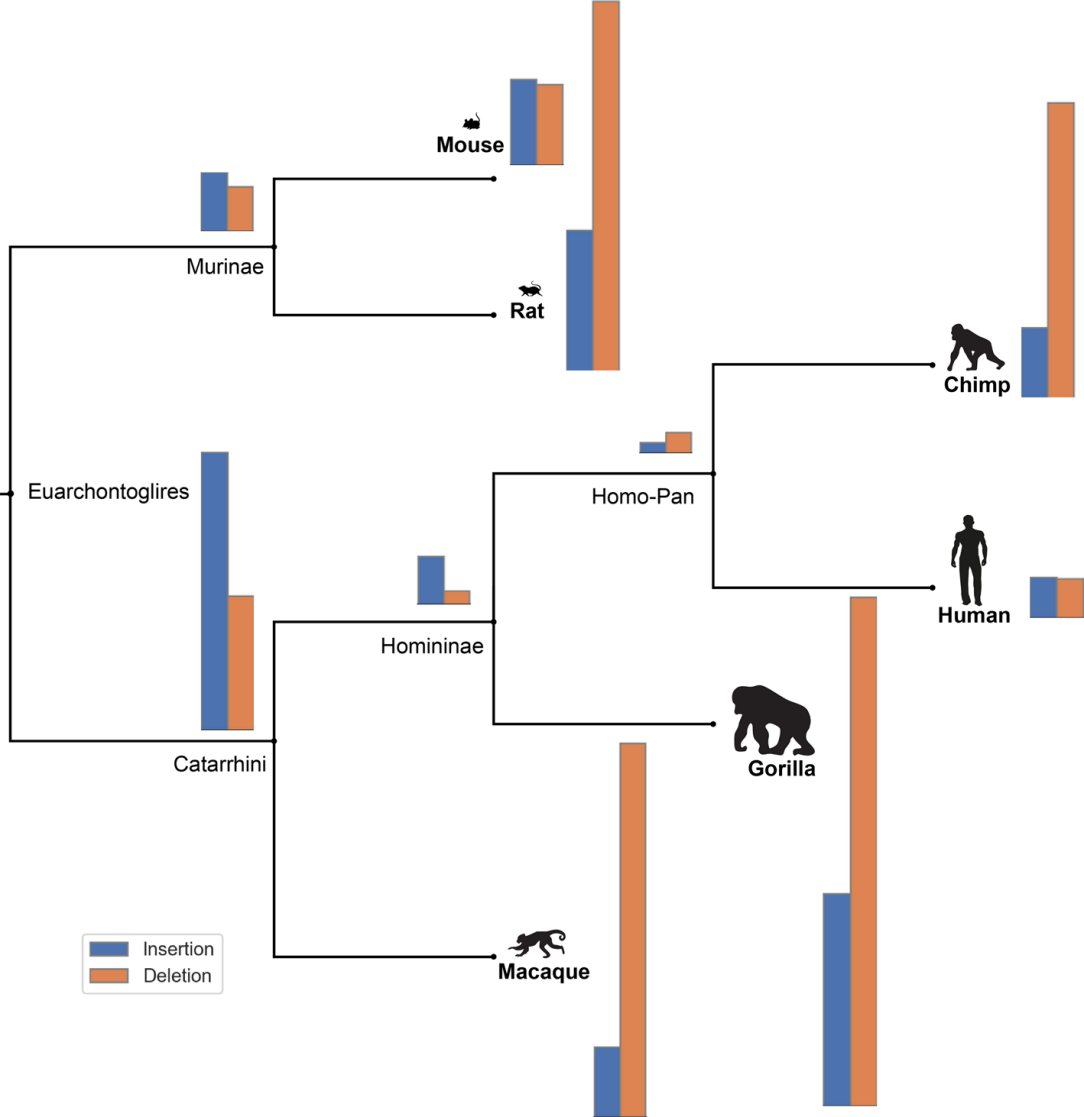
Total numbers of indel events per lineage across all datasets of the studied species overlayed on the species cladogram. *Gorilla* has the largest number of indel events per lineage while *Homo-Pan* and *Homininae* have the lowest number of indel events, respectively (see Table 1)

238 of orthologous groups had no gap characters in the inferred MSAs, presumably due to strong conservation. These groups were therefore excluded from the indel statistics presented here. For the remaining $3'668$ orthologous groups, the total numbers of inserted and deleted residues on the species tree are visualized in Fig. 1, and more detailed statistics are presented in Table 1. The *human* lineage had the lowest number of inserted and deleted characters, as well as overall gap characters in the sequences ($4.01\%$ of total sequence length). This is strongly contrasted by the *rat* lineage, which experienced the highest indel numbers among all studied species with $5.92\%$ of its total sequences in MSAs consisting of gap characters. The *macaque* and the *gorilla* lineages also had a higher number of gaps in their sequences, with $5.71\%$ and $5.42\%$, respectively. These two primate lineages (i.e. *macaque* and *gorilla*) also had the longest average gap stretch lengths (on average 18.2 amino acids for *macaque*, 15.2 for *gorilla*), compared to *human* (11.5) and all other lineages. *Homo-Pan* ancestral lineage experienced the lowest number of inserted and deleted residues, although this can be expected since this lineage corresponds to the shortest branch length on the species tree.

Next, we calculated the insertion–deletion bias as the ratio between the numbers of insertion and deletion events (see Fig. 15 in Appendix). Overall, the number of deletions was larger than the number of insertions for all six extant lineages except for the *human* and *rat*. The bias towards deletions was particularly strong in *macaque* (0.19) and *chimp* (0.24), but also well pronounced in *rat* and *gorilla* (0.38 and 0.42 respectively).

In contrast, most ancestral lineages displayed a bias towards insertions, which was particularly pronounced in the *Homininae* (3.39) and *Catarrhini* ancestors (2.07). This effect could be explained in several ways. PIP, like the most commonly used substitution models, assumes that the evolutionary process is at equilibrium. In particular, PIP assumes that the average expected sequence lengths at the root and the tips are the same. If this assumption is violated and the sequence length at the root is shorter, it may have to be balanced out by an increased insertion rate near the tree’s root, making this a dataset artifact. This effect could also be an artifact of the model. The included simulation study shows that a slight insertion bias is detected on simulated data. However, the effect is small and would not be able to explain the full extent of the bias we observe here. Lastly, this could be part of the true dynamics of insertions and deletions through time.

**Table 1:** Summary statistics of gaps and indels on mammalian data

Lineage/Clade	Gap characters	Average gap length	Total # of gaps	% gap characters	Average branch length	Ins	Del	Ins-Del bias
***Human***	$103'189$	11.5	$11'252$	4.01	0.004	$4'900$	$4'708$	1.04
***Chimp***	$130'673$	11.5	$11'546$	5.14	0.006	$8'647$	$35'939$	0.24
*Homo-Pan*								
*(Human, Chimp)*	$103'381$	11.5	$11'237$	4.02	0.002	$1'411$	$2'645$	0.53
***Gorilla***	$137'499$	15.2	$10'937$	5.42	0.014	**25’353**	**60’705**	0.42
*Homininae*								
*(Human, Chimp, Gorilla)*	$102'147$	11.5	$11'285$	3.97	0.015	$5'976$	$1'761$	**3.39**
***Macaque***	$144'537$	**18.2**	$11'613$	**6.8**	0.021	$8'855$	$47'030$	0.19
*Catarrhini*								
*(Human, Chimp, Gorilla, Macaque)*	$106'362$	11.5	$11'545$	4.14	0.109	$33'191$	$16'062$	**2.07**
***Mouse***	$121'087$	7.1	$14'417$	4.74	0.041	$10'664$	$10'025$	1.06
***Rat***	**149’525**	7.4	**14’802**	**5.92**	0.045	$17'191$	$44'990$	0.38
*Murinae*								
*(Mouse, Rat)*	$121'726$	7.4	$14'703$	4.77	0.092	$7'303$	$5'538$	**1.32**

The bold numbers reflect the parameter’s lower and upper bounds

#### Tracing sequence lengths along the tree

**Fig. 2:**
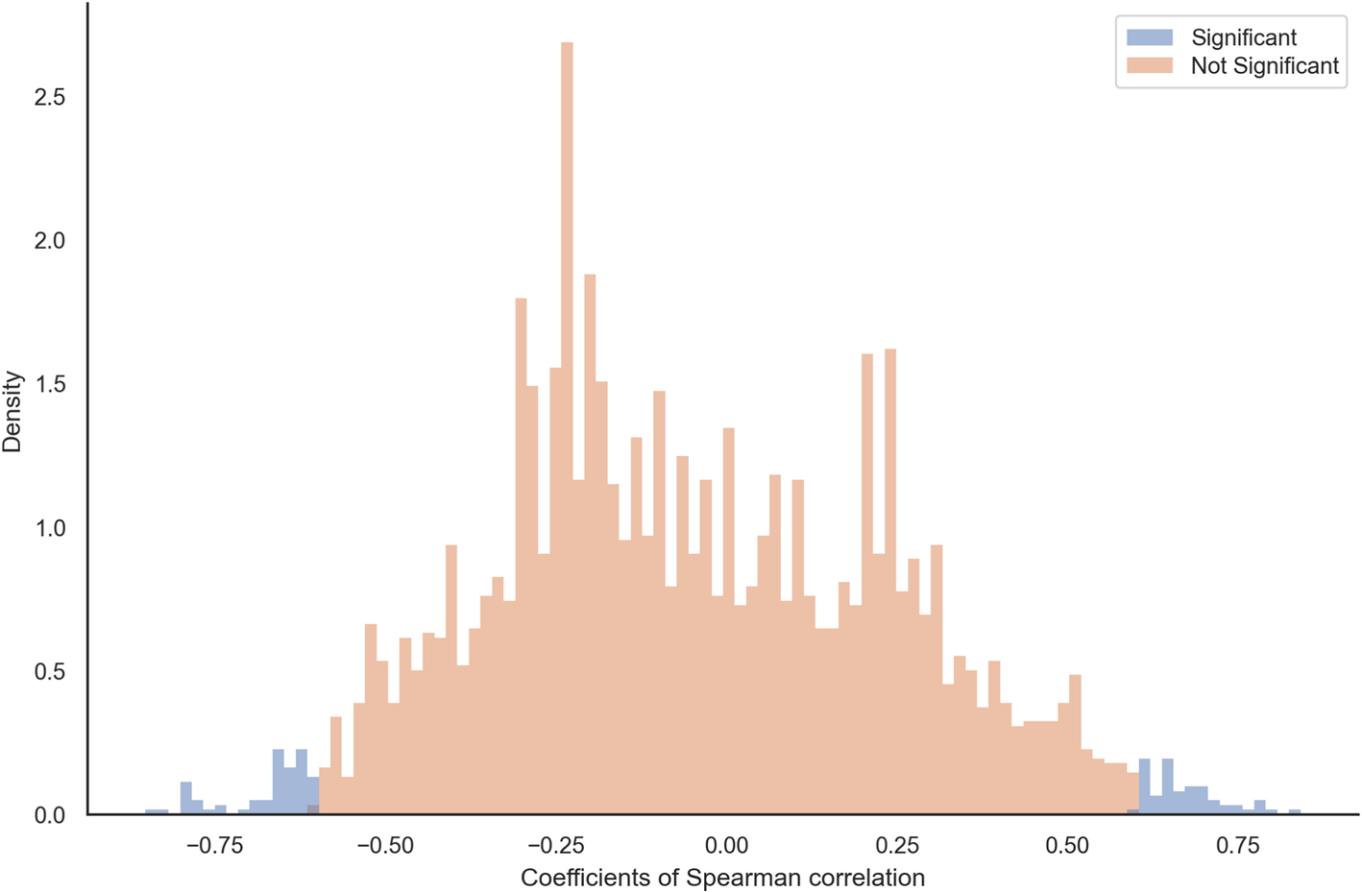
The distribution of Spearman correlation coefficients between sequence length (at the tips and root) and branch lengths per OMA groups on six mammalian species

Further, we investigated whether the observed deletion bias in extant lineages affects the sequence length dynamics across the species phylogeny. For each orthologous group, we computed Spearman correlation coefficients between sequence lengths (observed at the leaves or inferred at the ancestral nodes, gap characters removed) and the evolutionary distance (i.e., branch lengths). The majority of analyzed orthologous groups showed no significant correlations at a $5\%$ significance threshold. Nevertheless, we observed significant correlations in $3.46\%$ of orthologous groups with positive correlations for 59 genes and negative correlations for 68 genes (Fig. 2). This suggests that $1.85\%$ of analyzed gene sequences had the tendency to shrink, while $1.61\%$ had shown a tendency to grow. However, if we apply conservative correction for multiple testing by setting the individual *p*-values to $0.05/3'688$, we see no significant correlations.

#### Gap length distribution preserved over time

We asked whether the gap distributions in the six observed sequences differed from those in the inferred ancestral sequences. The gap distribution in the inferred MSA of the six observed sequences results from the PRANK alignment and would, therefore, exhibit any inherent systematic biases of the PRANK method, if any. By analyzing whether a change in gap length distribution occurs at the inferred ancestral sequences, we aim to evaluate whether ARPIP tends to bias the distribution in a given alignment towards shorter gaps.

**Fig. 3:**
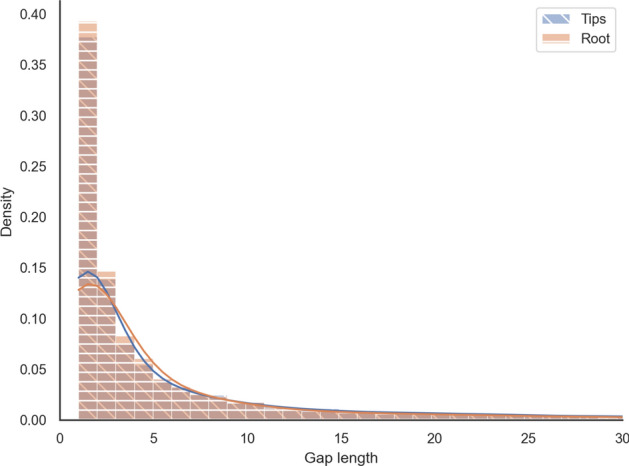
The empirical gap length distribution of tips vs. root on mammalian sequences. The plot is a histogram with 100 bins cut off at a gap length of 30 residues to eliminate the uninformative tail

Such an effect is expected to be maximal at the “centre” of the tree, corresponding to the midpoint root, where the tree height and, consequently, the uncertainty is the largest. In more than $96\%$ of our datasets, the midpoint and the evolutionary roots are the same. Therefore, we compared the empirical distribution of gap lengths at the root with the distribution at the leaves over all analysed OMA groups. The Kolmogorov-Smirnov two-sample test fails to reject the hypothesis that both were sampled from the same underlying distribution at the 0.05 significance level (*p*-value $\approx 0.11 > 0.05$). The two distributions are depicted in Fig. 3.

**Fig. 4:**
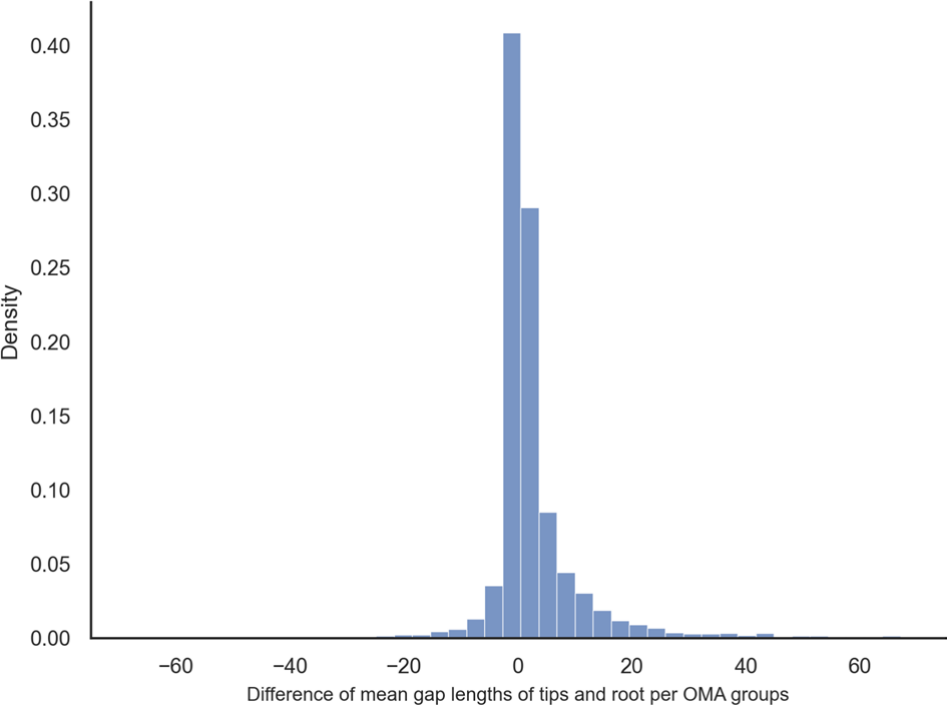
Paired difference of mean gap lengths per OMA groups on mammalian data (with 100 bins)

Furthermore, for each OMA group, we computed the mean gap lengths at the root and the mean gap lengths at the tips. The differences between the means are distributed around zero with a heavier tail in the positive range, which leads to an average difference of 3 characters, meaning that gaps at the tips tend to be around 3 characters longer (Fig. 4).

#### Inserted segments are longer than deleted segments

**Fig. 5:**
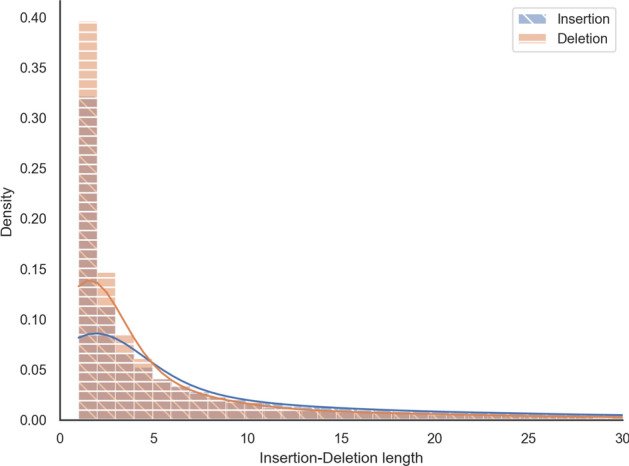
The empirical distribution of inserted vs. deleted segment lengths. The plot is a histogram with 100 bins cut off at a gap length of 30 residues to eliminate the uninformative tail

Finally, we compared the empirical distributions of multiple-character insertion and deletion events over time on the phylogeny. Figure 5 depicts that the empirical distributions of insertions and deletions are consistent with the empirical gap length distribution as single-character events are the most frequent, and their frequency decreases as the length of the event increases. However, the Kolmogorov-Smirnov two-sample test rejects the hypothesis that the insertion and deletion lengths follow the same underlying length distribution at the 0.05 significance level (*p*-value $\approx 1.6e^{-05} < 0.05$). This indicates that modelling insertion and deletion lengths separately is more meaningful than assuming the fragment lengths have the same distribution. We also observed that insertions tend to be significantly longer than deletions; the mean insertion length was 14.64, while it was 7.75 for deletion events.

### Results on simulated data

To study ARPIP under fully controlled conditions, we have simulated sequences with INDELible. To set realistic parameters, we sampled $1'000$ random OMA groups. For each sampled OMA group, we used the corresponding PhyML tree to evolve a replicate on it, with the root sequence length of $1'000$ amino acids, indel rate of 0.1, and indel lengths distributed according to the Zipfian distribution with exponent 1.7. INDELible’s maximum indel length parameter was set to the length of the longest gap in the PRANK MSA of the OMA group in question. We supplied the true simulated MSA of the observed sequences to ARPIP for all the analyses.

#### Reconstruction accuracy

**Fig. 6:**
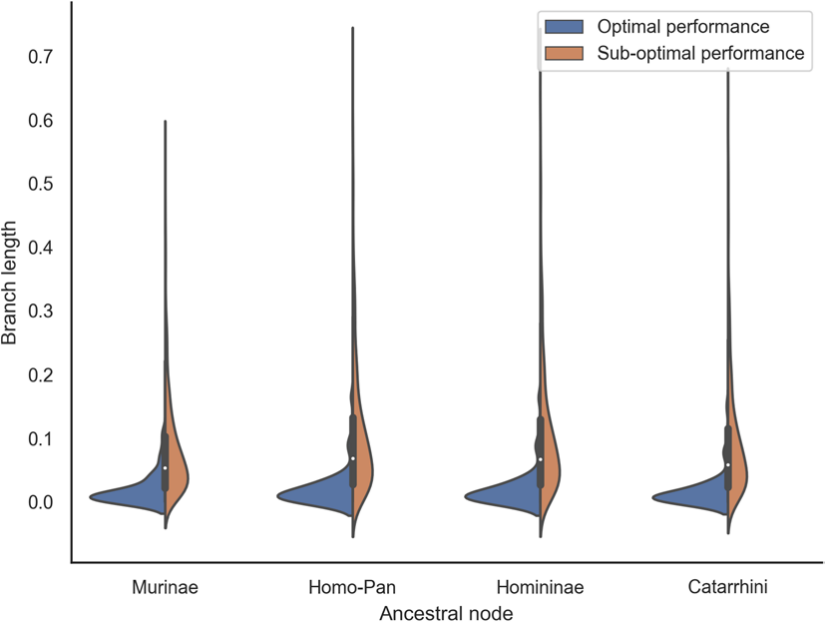
Distribution of ancestral node branch lengths in the simulated data, grouped by inference performance

**Fig. 7:**
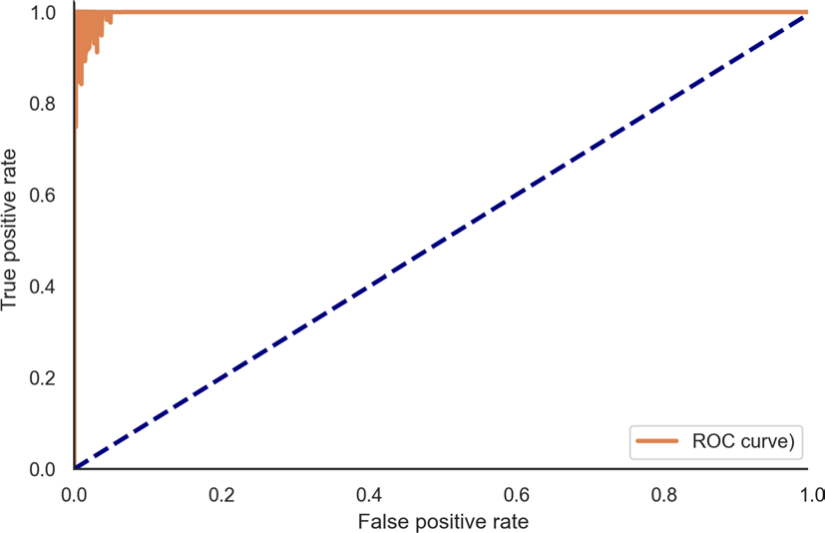
ROC curve: true positive (recall or sensitivity) vs. false positive (1-specificity) rates at the ARPIP gap estimation

On simulated data, ARPIP inferred a positive insertion–deletion bias in all nodes of the trees; i.e., more individual characters were inserted than deleted (Appendix Fig. 16). It correctly reconstructed more than $98\%$ of ancestral residues, resulting in $90\%$ correctly inferred ancestral columns (Appendix Table 2). The average precision^1^ in gap character inference was $94\%$, with a recall^2^ of $97\%$. We classified the simulation results according to the F-score (a measure of predictive performance defined as the harmonic mean of precision and recall) in gap retrieval into “optimal” (132 samples with F-score $\ge 99\%$) and “sub-optimal” (851 samples with $\le 70\%$ F-score $< 99\%$). Figure 6 shows the branch length distributions for the two classes. The optimal samples tended to have shorter branches. For these samples, we observed a higher accuracy in gap reconstruction. Indeed, shorter branches provide more information, and we expect lower variances and higher accuracy. In contrast, longer branches and higher evolutionary distances show lower accuracy, potentially due to the evolutionary signal becoming saturated. Furthermore, the insertion probability in PIP is proportional to branch lengths. Thus, the choice of insertion points also depends on the relative branch lengths of the phylogeny. Figure 7 shows the ROC curve points for each sample (and not just one point, i.e. the average).

#### Tracing sequence lengths along the tree

**Fig. 8:**
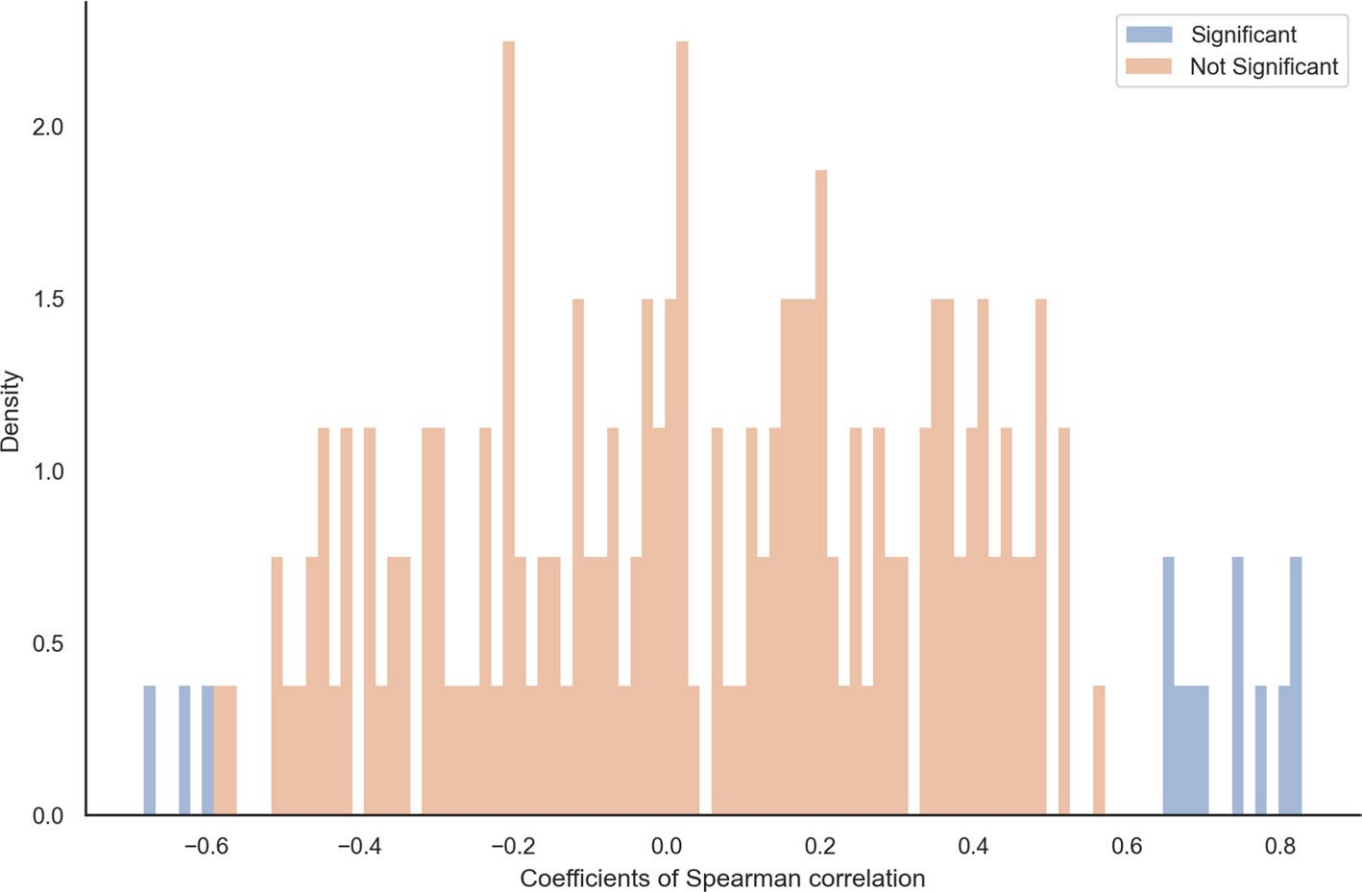
The distribution of Spearman correlation coefficients between sequence length (at the tips and root) and evolutionary distance per OMA group on simulated data

Analogous to the real data analysis above, we correlated the sequence length without gaps in each node with the node’s branch length for each replicate. Again, the majority of the Spearman coefficients were not significant at the threshold of 0.05. Among the $7.91\%$ significant ones, we observed 11 positive and 3 negative correlations (Fig. 8). Contrary to the real data, here, the majority of the significant replicates tended to grow, while $0.3\%$ were shrinking. This is consistent with the positive indel bias.

#### Gap length distribution is preserved over time

**Fig. 9:**
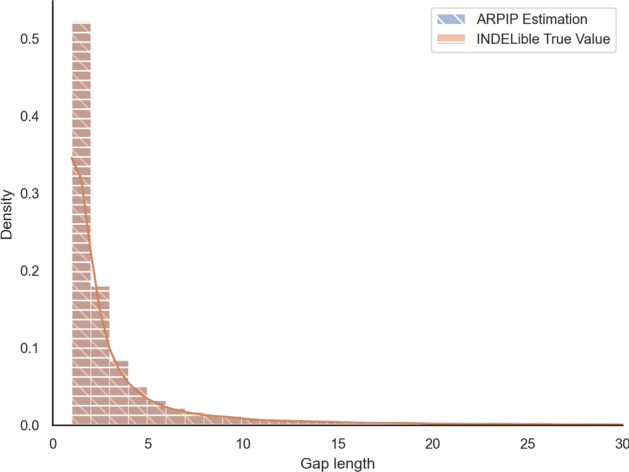
Overlapped distributions of gap lengths from ARPIP inference and INDELible true values. The plot is a histogram with 100 bins cut off at a gap length of 30 residues to eliminate the uninformative tail

**Fig. 10:**
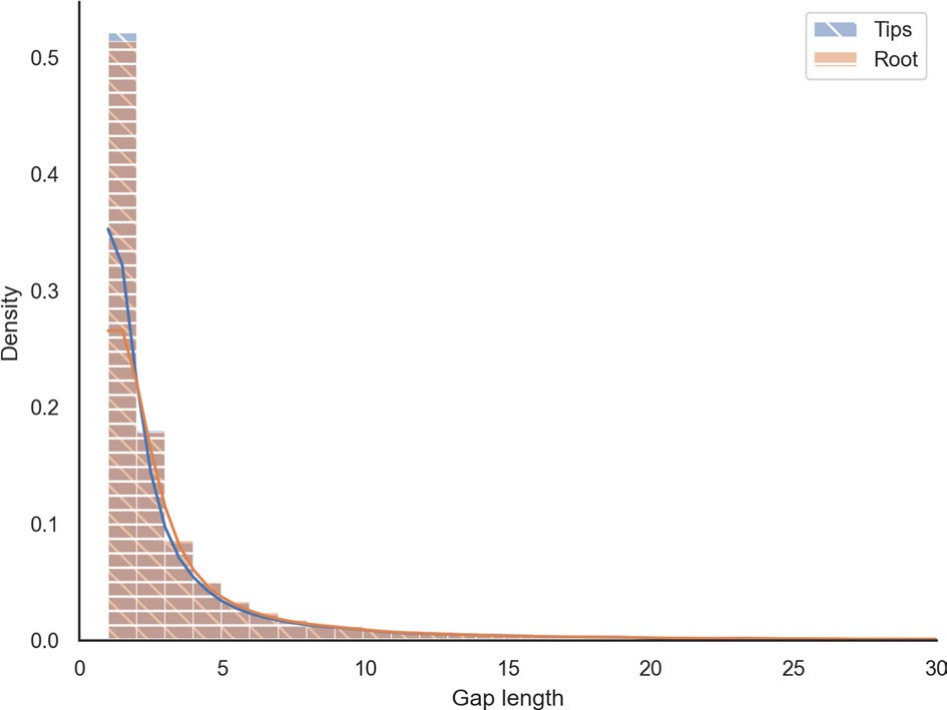
Empirical gap length distribution at the tips vs. the root in simulated sequences as a histogram with 100 bins cut off at a gap length of 30 residues to eliminate the uninformative tail

Next, we asked if the gap length distribution in the inferred ancestral sequences differed from the true distribution, i.e. the one generated by the simulation. The Kolmogorov-Smirnov test of the two distributions has the *p*-value of $0.99 > 0.05$, failing to reject that the distributions are the same (Fig. 9). According to the PIP model, we expect sequence lengths to be preserved, meaning neither shrinking nor growing. Furthermore, there seems to be no decline of gap lengths towards the root of the tree, as the gap length distribution inferred at the root of the tree matches the distribution in the observed sequences at the leaves (Fig. 10), Kolmogorov-Smirnov test with *p*-value of $0.702 > 0.05$. Note that in contrast to the real data case above, where the gaps at the leaves were inferred by PRANK, here we were able to compare to the true (simulated) MSA.

**Fig. 11:**
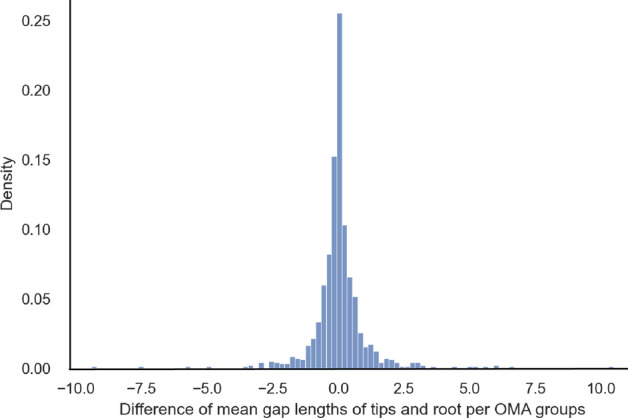
Paired difference of mean gap lengths per OMA groups on mammalian data (with 100 bins)

To further quantify the difference between simulated and inferred distributions, we computed the mean gap lengths at the root and the mean gap lengths at the tips for each of the 1000 replicates. The differences between the means were symmetrically distributed around zero (Fig. 11). The differences were not statistically different from zero (Mann–Whitney test, $p=0.67$; two-sample t-test, $p=0.997$).

In summary, our simulation findings corroborate the results from real data. ARPIP preserves the gap lengths from the input alignment.

#### PRANK alignments preserve the gap length distribution on simulated data

To reduce uncertainty in our analyses, we ran a simple test to determine whether PRANK alignments of simulated data preserve the length distribution of gaps at the leaves of the tree. Given the unaligned sequences from INDELible, we have aligned them with PRANK using the correct guide tree and compared the gap length distribution of the true alignment produced by INDELible vs. the alignment produced by PRANK. Unfortunately, due to the change in the MSA, we do not have the exact simulated ancestral sequences and are thus unable to evaluate the precision of ancestral state reconstruction.

**Fig. 12:**
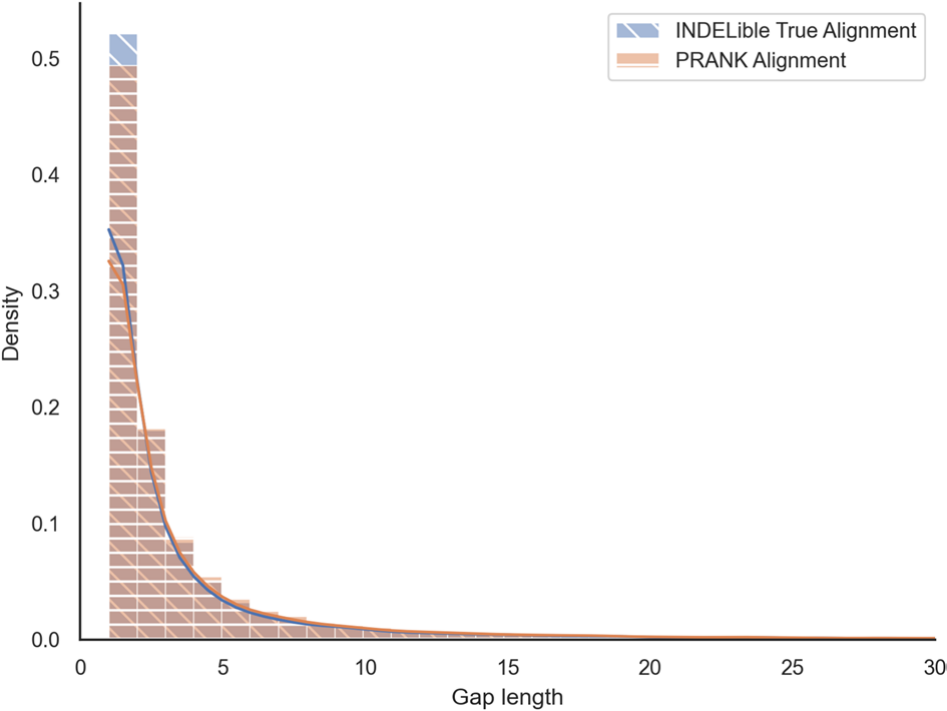
Empirical gap length distribution at the tips of the tree in the true simulated MSAs and the MSAs inferred by PRANK as a histogram with 100 bins cut off at a gap length of 30 residues to eliminate the uninformative tail

Using the Kolmogorov-Smirnov two-sample test, we get a *p*-value of $0.99 > 0.05$, meaning that the two distributions are not significantly different (Fig. 12). This is a good sign that, at the very least, on simulated data, PRANK realigns the sequences well and does not introduce any bias. This is insufficient proof that there is no other bias that could show up in real data, but it is a good indicator nonetheless.

## Discussion and conclusions

Until recently, state-of-the-art ASR methods focused on inferring ancestral characters. Indels were often mishandled – either by removing gappy MSA columns, treating gaps as ambiguous characters [32], or reconstructing ancestral gaps with ad-hoc indel methods like “indel coding” [21]. Further, such methods typically do not easily distinguish between insertions and deletions. Unlike previous approaches, ARPIP reconstructs insertions and deletions independently and uses the evolutionary indel model PIP. However, PIP only describes single-character indels.

In contrast to ASR, methods for MSA inference are more advanced with respect to allowing for long indels. One of the most advanced aligners is PRANK; it uses the phylogeny to distinguish insertions from deletions and, thus, infers phylogenetically meaningful long indels. All current ASR methods take an MSA as input. Here, we have shown on real data (with PRANK alignments) and by simulation (with the true simulated MSAs from INDELible) that the ancestral estimates by ARPIP preserve the long indel structure present in the MSA. This surprising result can partly be explained by the fact that under PIP the insertion and deletion points of a site only depend on the gap patterns (i.e. the presence and absence of gaps), and are independent of the character states [15]. Neighboring sites with identical gap patterns form long indels and lead to identical indel histories (see, for example, Appendix B). Further studies will be needed to quantify how differences in neighboring gap patterns affect long indel preservation. Based on ARPIP’s strong performance, we hypothesize that minor pattern differences will still preserve most long indels.

Furthermore, in line with the biology [33] and previous bioinformatics studies [26, 31, 34], we found that deletions are more frequent than insertions in extant lineages. Such deletion bias has been detected across the whole tree of life and has multiple possible evolutionary explanations. For example, He et al. [30] suggest that even strictly balanced insertion and deletion rates result in a linearly increasing genome size through time rather than a completely fixed genome size. The authors attribute this effect to the fundamental asymmetry of indels, which can be attributed to the inherent difference in how the two mechanisms change the size of the genome. An insertion creates an additional character, which in turn creates more opportunities both for other insertions and deletions by adding another site where events can happen. In contrast, a deletion removes opportunities for events to happen as the number of characters is reduced. The authors suggest that while the huge variety in genome sizes among species seems to require exponential size growth, the effective insertion bias cannot act for prolonged periods of evolutionary time. Consequently, the mechanisms producing larger genome sizes only act sporadically and are likely to be removed in the long term, making them very difficult to detect by looking into existing genomes. On the other hand, the commonly detected deletion bias could be an artifact of inference. A similar effect, “pull-of-the-present,” is well known in phylodynamics, where younger lineages show seemingly higher birth/lower death rates, even though the real rates remain the same [35]. This effect stems from the fact that we are observing a snapshot of evolutionary history that is cut off from the future, meaning that while some of the present-day lineages might go extinct, they have had less time to do so than older lineages and thus are more likely to have been sampled. In the case of a universally observed deletion bias, it could mean that deletions might appear more frequently in the present sequences because the deleterious deletions have not yet been removed by selection.

Finally, until now, virtually all studies on indel length distributions have lumped the insertions and deletions together, often just inferring gap length distributions. There are a few notable exceptions, for example, Tanay and Siggia [36]. These studies, while insightful, are not general-purpose and are limited to a restricted set of organisms as they require extensively annotated and closely related genomes. In contrast, our approach allows us to quantify insertion and deletion processes and length distributions on any MSA of interest. As a step forward, we suggest inferring separate distributions for insertion and deletion lengths. Our findings from mammalian data strongly point to longer insertion lengths than deletion lengths. Further, given the higher prevalence of deletions and the remarkable uniformity of protein length distribution across the tree of life [37], it is conceivable that the two distributions differ, with deletions lengths having a smaller mode than insertions. Recent work from Tal Pupko’s lab is a notable step in the direction of inferring indel length distributions based on event reconstruction [38].

## Data and methods

### Sequence acquisition and alignment

**Fig. 13:**
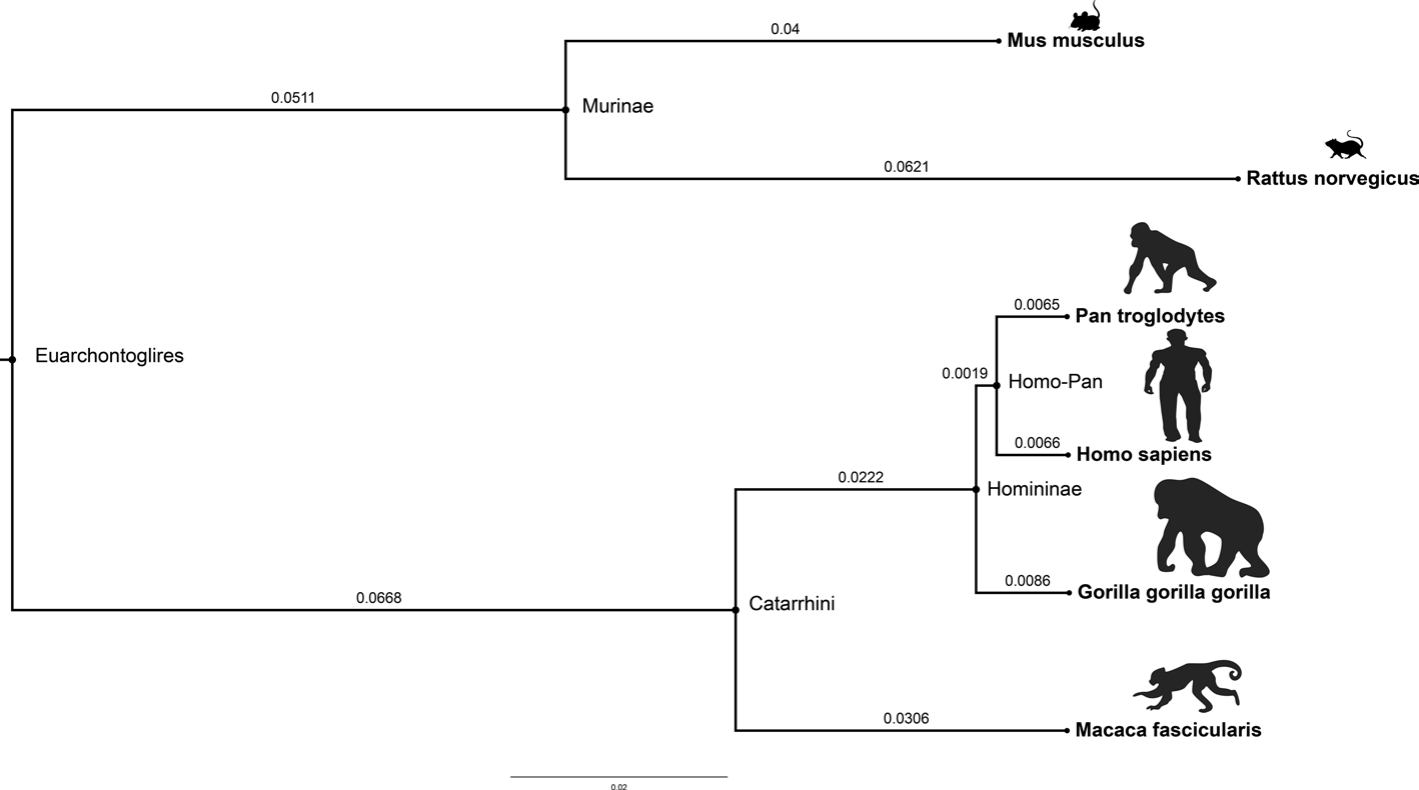
Illustration of the guide tree extracted from 43 *eutherian* mammals. The branch lengths were estimated using pairwise MSA in Ensembl Compara v.105

**Fig. 14:**
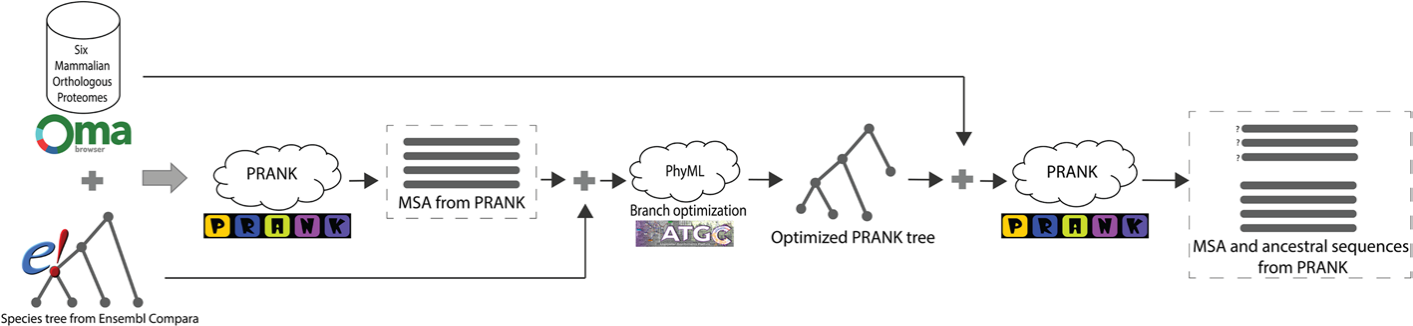
Data acquisition pipeline

**Fig. 15:**
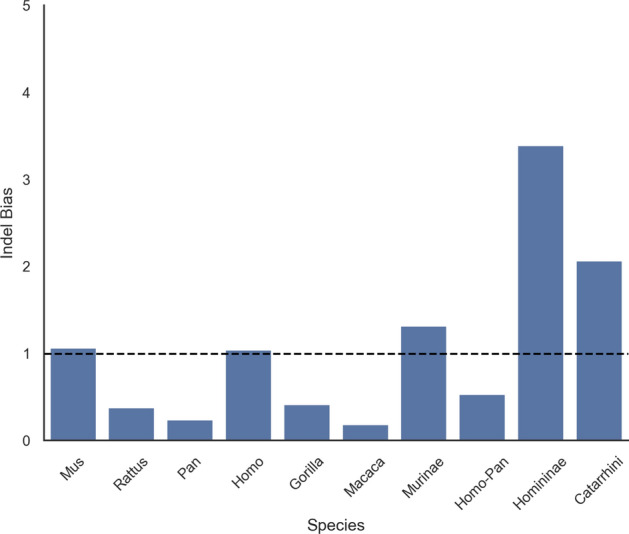
Indel bias (ratio of insertion to deletion events) in mammalian data. A ratio of less than one indicates a bias toward deletions

First, we used the OMA database [24] to obtain orthologous protein sequences so that each orthologous group (OMA group) contained one sequence from each of six mammalian species, namely *human*, *chimp*, *gorilla*, *macaque*, *mouse*, and *rat*. The OMA database is known for its higher precision but lower recall compared with the majority of other methods [24, 39]. A corresponding species tree was extracted from the Ensembl Compara v. 105 [40] by pruning a larger mammalian tree to the six species considered in this study (see Fig. 13). This species tree was then provided as a guide tree for reconstructing multiple sequence alignments (MSAs) using PRANK+F, a phylogeny-aware progressive aligner distinguishing insertions from deletions [23]. For each reconstructed MSA, we estimated gene trees with branch lengths by maximum likelihood with PhyML v3.3.20211231 [41]. We then filtered out the datasets for which the gene trees matched the species tree. Finally, a refined PRANK MSA was inferred for each orthologous group using a species tree with re-optimized branch lengths as a guide tree (see Fig. 14 for the flowchart showing the pipeline). The WAG amino acid substitution model [42] was used in all analysis steps, including the ancestral sequence reconstruction described below.

### Ancestral sequence reconstruction

The refined MSA was used to infer ancestral sequences at all species tree nodes with optimized branch lengths with our recent method implemented in ARPIP [15]. Evolutionary changes on a phylogeny are described via the PIP model [11], where insertions follow a Poisson process, while substitutions and deletions follow a continuous-time Markov model with an absorbing state. The ARPIP method includes two main steps. First, the method infers the most probable indel scenario on a given phylogeny, independently for each MSA column. Next, similar to FastML [43], ancestral characters are reconstructed on a subtree of the given phylogeny obtained by pruning it to the inferred indel scenario. For ASR analyses, the root was placed on the internal branch connecting the *Catarrhini* and *Murinae* clades. Then, midpoint rooting was used to define the location of the root on this branch.

### Simulating data

We simulated 1’000 data sets with INDELible [44]. To set realistic parameters, we sampled uniformly at random 1’000 OMA groups and extracted the corresponding PRANK MSAs and species trees with PhyML-optimized branch lengths (as described above). For each sample, we simulated a replicate on the PhyML tree using a sequence of 1’000 amino acids at the root. We use a Zipfian indel length distribution with $\alpha =1.7$, a maximum indel length equal to the maximum gap length of the OMA group in question, and an indel rate of 0.1. Sequence lengths in the simulated samples ranged between 336 and 1’730 amino acids, while the gap lengths ranged from 1 to 1’451 characters. Around $1\%$ of simulations produced biologically unrealistic sequences with extremely long gaps, for example, the sample with a 1’451 character long gap. Such samples would be considered noisy in real datasets (possibly due to sequencing errors) and were thus also removed from the simulation analysis before evaluating reconstruction performance. Only four simulated samples contained no gaps at all and were also removed from analysis. The final simulated dataset contained 786 to 1’371 amino acid long sequences and the gap lengths ranged from 1 to 235 characters.

We provided the true MSA from the simulation and the PhyML tree (i.e. true tree) to ARPIP for ancestral reconstruction.

## Figures and Tables

**Fig. 16:**
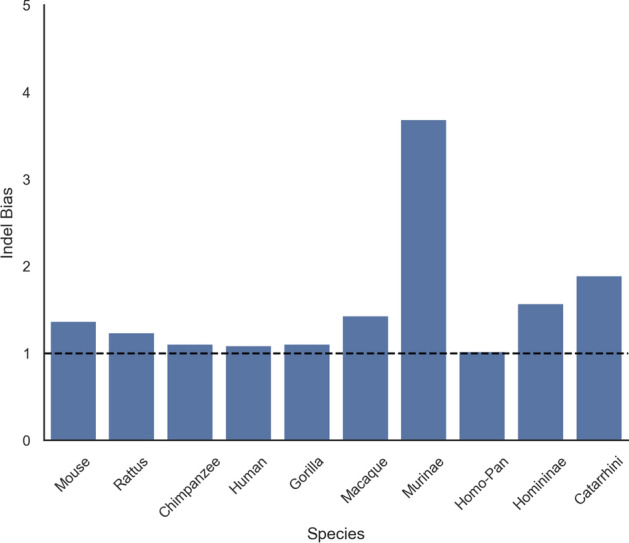
Indel bias (ratio of insertion to deletion events) in simulated data. A ratio of less than one indicates a bias toward deletions

**Fig. 17:**
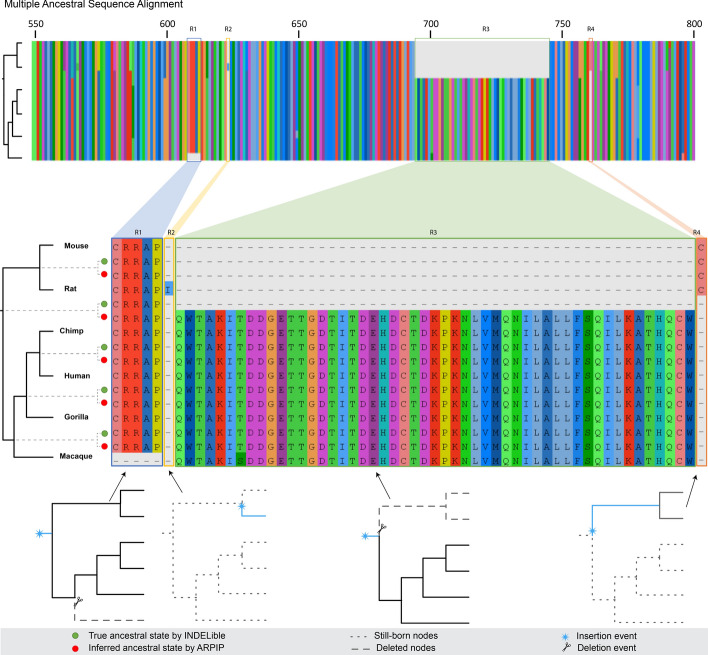
Multiple ancestral sequence alignment of ARPIP inference and INDELible true ancestral states for sites $550-800$ of sample $s_1$. The indel inference for each site is shown at the bottom of the figure

**Fig. 18:**
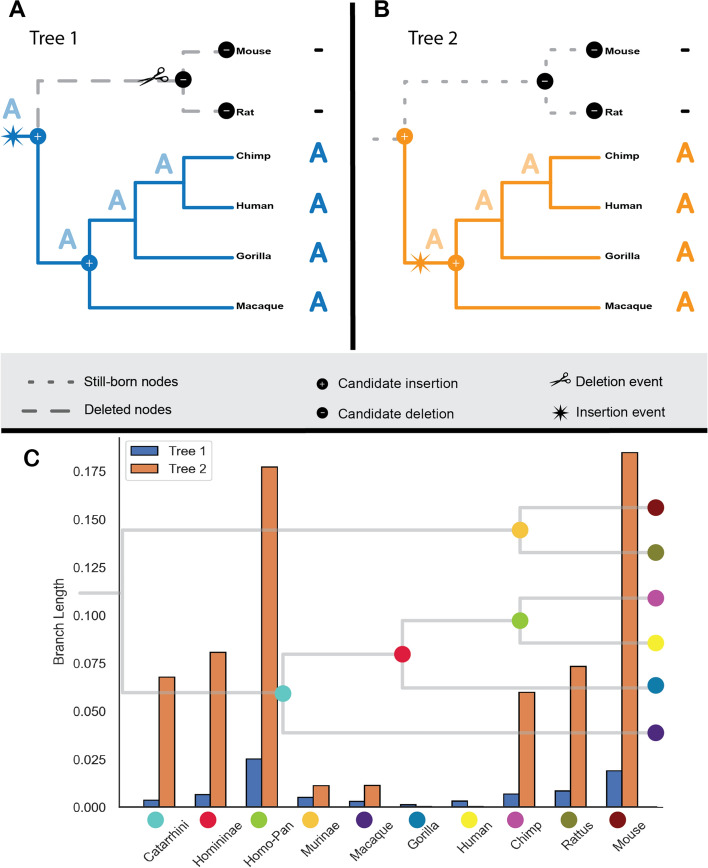
A, B) Two different indel scenarios for a single MSA with various branch lengths. C) Histogram of branch lengths of two selected simulated samples

**Fig. 19:**
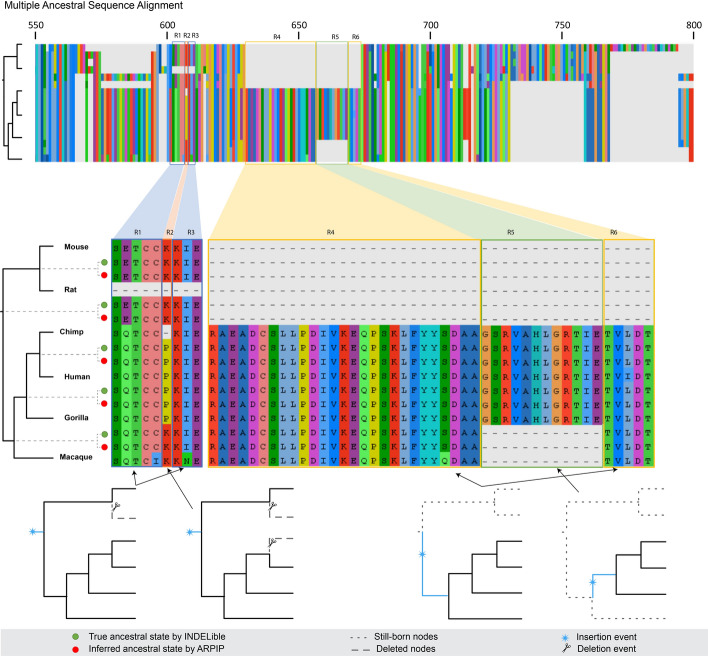
Multiple ancestral sequence alignment of ARPIP inference and INDELible true ancestral states for sites $550-800$ of sample $s_2$. The indel inference for each site is shown at the bottom of the figure

## Data Availability

This manuscript is accompanied by the scripts used to produce the results. The experimental data used in this manuscript is freely available from https://doi.org/10.5281/zenodo.10798097. The Python scripts used for data processing and analysis are also available at https://github.com/acg-team/single-char-indel-ASR-preserves-long-indels.

## Appendix A Tables and figures

### Tables related to the accuracy of reconstruction on the simulated data

We report the average accuracy over all the samples (See Tables 2, 3).

**Table 2:** ARPIP performance in simulation. All metrics include the root sequences and have been computed for each sample individually. We report the averages over the samples

Metric	Consistency (%)
Proportion of correctly inferred ancestral characters	$97.88 \pm 2.01$
Proportion of correctly inferred ancestral columns	$90.35 \pm 2.01$
Proportion of correctly inferred ancestral amino acids (i.e., excluding gaps)	$97.75 \pm 2.55$
Gap precision	$94.27 \pm 5.37$
Gap recall (sensitivity)	$96.99 \pm 3.97$
Gap F-score	$95.46 \pm 3.37$
Gap specificity	$99.29 \pm 1.17$

**Table 3:** ARPIP performance in gap character inference by simulation. Performance is shown individually for each internal node

Lineage/Clade	Gap consistency/accuracy (%)		
	Precision	Recall	F-score
Murinae	$98.74 \pm 5.24$	$99.93 \pm 1.10$	$99.31 \pm 1.60$
Homo-Pan	$99.99 \pm 0.11$	$99.9995 \pm 0.02$	$99.9970 \pm 0.06$
Homininae	$99.98 \pm 0.20$	$99.94 \pm 1.51$	$99.95 \pm 0.97$
Catarrhini	$98.48 \pm 1.75$	$99.96 \pm 0.60$	$99.71 \pm 0.99$
Euarchontoglires	$77.71\pm 17.22$	$85.49 \pm 17.46$	$79.44\pm 15.07$

### Indel bias plots for the mammalian and simulated data

See Figs. 15, 16.

## Appendix B Study of example reconstructions on simulated data

To get a better intuition for the performance of indel reconstruction under PIP, we have selected two samples from the pool of simulated data for closer examination. The sample $s_1$ is among the data with the lowest gap retrieval performance, while the second sample $s_2$ is a sample with a relatively good gap retrieval score.

### Sample 1: Sub-optimal performance

We have selected two samples from the pool of simulated data to study the performance of gap reconstruction of ARPIP. Sample $s_1$ is among the samples with the lowest F-score. For $s_1$, the F-score is $72.86\%$, while precision and recall are $100\%$ and $57.31\%$, respectively. This means that all the inferred gaps were correct, but only around half of the gap characters were inferred. The inference accuracy at the root was the lowest not only in this sample but also in all the samples from the simulated dataset (see Table 3). Figure 17  visualizes a segment of $s_1$ to investigate ASR performance and gap patterns.

Figure 17  highlights the inferred and true ancestral sequences for four regions of interest. Region R1 depicts five independent insertion and deletion events. Each insertion happened at the root, followed by deletion at the *macaque* taxon. Region R1 does not affect the ancestral gap length distribution, but this typical case happens for a single stretch of gaps at the taxa node. Similarly, region R2 occurs when a single residue in an MSA column exists. A single insertion at the taxa node usually represents a single residue insertion event. This inserted site will show up as a gap in all ancestral nodes, affecting the gap length distribution at the ancestral node, while in reality this site never existed in the ancestor.

Region R3 contains multiple long gaps within both ancestral and taxa species. In this case, as the neighboring site across both ancestral and descendent nodes has the same gap pattern, ARPIP infers the same indel scenario given fixed model parameters. A single insertion at the tree’s root is followed by deletion at the *Murinae* branch.

In $s_1$, the gap reconstruction accuracy in the root node is very low due to low recall, meaning that ARPIP reconstructs a small fraction of the gaps in the root node. The cause for low gap character retrieval rates remains to be explained. Figure 18 shows different indel scenarios for a constant MSA column with respect to the branch length of the tree.

Region R4 is a masking indel event of region R3, as we have an insertion event at the branch leading to the node *Murinae*. This is a single-site indel event affecting the ancestral and descendant gap distribution. Notice that we have a single insertion at node *Murinae* without any deletion events.

### Sample 2: Optimal performance

In addition, we have selected sample $s_2$ with an overall F-score of $91.36\%$, resulting from $84.10\%$ precision and $100\%$ recall. This implies that all the gaps were inferred correctly, while a fraction of non-gap characters were falsely inferred as a gap. Figure 19 illustrates that ARPIP performs well in inferring ancestral sequences despite the complex gap pattern, with $93.42\%$ overall reconstruction accuracy. Moreover, sample $s_2$ performs relatively well at the root node compared to sample $s_1$. Figure 19 shows the gap pattern in two selected neighboring regions (R1-3) and (R4-6).

The PIP model tends to place the insertion events at the root because the Poisson process initiates at the tree’s root. Regions R1 and R3 have a repeated insertion at the root followed by a single deletion event at the *rat* taxon. A neighboring region denoted by R2 has an additional gap between the regions mentioned. ARPIP can adapt the indel event for this specific site while preserving the gap distribution for the other two regions. The gap pattern in these three regions did not affect the gap distribution of ancestral nodes.

The transition from gap pattern R1 to R2 (sites $606-607$) and R2 to R3 (sites $607-608$) suggests that introducing a new gap in another node would have minimal impact on gap inference. The transition from R4 to R5 (sites $656-657$) or R5 to R6 (sites $668-669$) shows the ARPIP can preserve gap distribution for the long ancestral gaps. The results suggest that ARPIP performs exceptionally on neighboring sites with long gaps but suboptimally at the root.

Neighboring segments R4–R6 show two different indel event patterns. We infer that the R4 and R6 segments have an insertion at the *Catarrhini* node, and the R5 segment has an insertion at *Homininae*, without any deletion events at these sites. These three neighboring regions would affect both the ancestral and descendant gap patterns. Like in sample $s_1$, region R5 separates R4 and R6 without negatively affecting the gap inference. This example shows that ARPIP is relatively good at preserving gap patterns in the neighboring sites.

## Footnotes

1 The percentage of correctly inferred gap characters among all inferred gap characters.

2 The percentage of correctly inferred gap characters among all true gap characters.
